# Identification and Evaluation of Recombinant Outer Membrane Proteins as Vaccine Candidates Against *Klebsiella pneumoniae*

**DOI:** 10.3389/fimmu.2021.730116

**Published:** 2021-10-20

**Authors:** Bao-Zhong Zhang, Danyu Hu, Ying Dou, Lifeng Xiong, Xiaolei Wang, Jingchu Hu, Shao-Zhen Xing, Wenjun Li, Jian-Piao Cai, Meiling Jin, Mengya Zhang, Qiubin Lin, Min Li, Kwok-Yung Yuen, Jian-Dong Huang

**Affiliations:** ^1^ Chinese Academy of Sciences (CAS) Key Laboratory of Quantitative Engineering Biology, Shenzhen Institute of Synthetic Biology, Shenzhen Institutes of Advanced Technology, Chinese Academy of Sciences, Shenzhen, China; ^2^ School of Biomedical Sciences, The University of Hong Kong, Hong Kong, Hong Kong, SAR China; ^3^ Department of Microbiology, The University of Hong Kong, Hong Kong, Hong Kong, SAR China; ^4^ Vaccine and Antibody Engineering, HKU-Zhejiang Institute of Research and Innovation (HKU-ZIRI), Hangzhou, China; ^5^ School of Chinese Medicine, Hong Kong Baptist University, Hong Kong, Hong Kong, SAR China; ^6^ State Key Laboratory of Emerging Infectious Diseases, The University of Hong Kong, Hong Kong, Hong Kong, SAR China

**Keywords:** *Klebsiella pneumoniae*, proteomics and bioinformatics, outer membrane proteins, vaccine, serotype-independent vaccines

## Abstract

*Klebsiella pneumoniae* found in the normal flora of the human oral and intestinal tract mainly causes hospital-acquired infections but can also cause community-acquired infections. To date, most clinical trials of vaccines against *K. pneumoniae* have ended in failure. Furthermore, no single conserved protein has been identified as an antigen candidate to accelerate vaccine development. In this study, we identified five outer membrane proteins of *K. pneumoniae*, namely, Kpn_Omp001, Kpn_Omp002, Kpn_Omp003, Kpn_Omp004, and Kpn_Omp005, by using reliable second-generation proteomics and bioinformatics. Mice vaccinated with these five KOMPs elicited significantly higher antigen-specific IgG, IgG1, and IgG2a. However, only Kpn_Omp001, Kpn_Omp002, and Kpn_Omp005 were able to induce a protective immune response with two *K. pneumoniae* infection models. These protective effects were accompanied by the involvement of different immune responses induced by KOMPs, which included KOMPs-specific IFN-γ-, IL4-, and IL17A-mediated immune responses. These findings indicate that Kpn_Omp001, Kpn_Omp002, and Kpn_Omp005 are three potential Th1, Th2, and Th17 candidate antigens, which could be developed into multivalent and serotype-independent vaccines against *K. pneumoniae* infection.

## Introduction

*Klebsiella pneumoniae* (*K. pneumoniae*) is a Gram-negative bacterium that is present in the natural flora of the human mouth and intestine. It mainly causes hospital-acquired infections, but in immunosuppressed individuals, it may also cause community-acquired infections (1). *K. pneumoniae* can cause several infections such as pneumonia, urinary tract infection (UTI), and liver abscess (2). Immunocompromised individuals, including people with diabetes, chronic lung conditions, HIV-positive people, and hospitalized patients, are the groups with the highest risk of *K. pneumoniae* infection (3). The World Health Organization (WHO) has expressed concern about *K. pneumoniae* developing antimicrobial resistance (AMR) (4). The WHO has listed *K. pneumoniae* as a member of the Carbapenem-resistant Enterobacteriaceae group of pathogens under the “critical” priority for R&D regarding new antibiotics (5). Extended-spectrum beta-lactamase (ESBL)-resistant strains have also been listed as a serious threat on the WHO list (5). Both carbapenemase-producing Enterobacteriaceae (CPE) and ESBL *K. pneumoniae* strains have been reported worldwide (5). CPE *K. pneumoniae* strains frequently also exhibit resistance to aminoglycosides and fluoroquinolones (5).

Alternative treatment options to *K. pneumoniae* are limited. Vaccines that are effective for these risk groups are highly desirable. Currently, although vaccines against *K. pneumoniae* have been explored, there are no commercial vaccines for the prevention against *K. pneumoniae*. Lipopolysaccharide (LPS, O-antigen) and capsular polysaccharide (K-antigen) are potential targets of *K. pneumoniae* vaccine, but they have obvious limitations. These antigens have been studied in detail, and 8 O-antigens and 77 K-antigens have been identified so far (6). O-antigens do not appear to be effective as *K. pneumoniae* vaccine targets because they cause toxic side effects in active immunization. K-antigens are immunogenic and non-toxic, but a vaccine must include at least 24 major K types to cover 70% of strains of *K. pneumoniae* (7).

Surface proteins are very important molecules that interact with the surrounding environment (8). They are involved in many biological processes including transport, intercellular recognition, and receiving and transmitting signals (9). These molecules play important roles in antibiotic resistance mechanisms and contribute to the virulence of the organism. They make up a diverse group of very important functions because they have a high probability of being recognized by the elements of the immune system and have the potential to become drug targets and vaccine candidates (10). However, no effective surface-protein-based vaccines have been developed for *K. pneumoniae*. We hypothesize that effective vaccine targets can be obtained by isolating and analyzing the surface proteins of *K. pneumoniae* to find suitable potential vaccine targets by using proteomics methods.

The aim of this study was to identify *K. pneumoniae* extracellular surface proteins by efficient and reliable second-generation proteomics and bioinformatics. The immunogenicity of selected potential *K. pneumoniae* outer membrane proteins (KOMPs) were tested in murine model. The protective efficacies of the candidate proteins were investigated in two *K. pneumoniae* infection models.

## Materials and Methods

### Bacterial Strains, Media, and Growth Conditions

The *K. pneumoniae 260* strain was provided by Professor Yigang Tong and isolated from the Respiratory Department of the 307 Hospital of the Chinese People’s Liberation Army. *K. pneumoniae 260* is resistant to a variety of antibiotics (Amikacin, Cefuroxime-Sodium, Ciprofloxacin, Piperacillin, etc.). *K. pneumoniae 260* strain was grown at 37°C in Luria–Bertani broth. Before bacterial challenge, overnight cultures of *K. pneumoniae 260* strain was sub-cultured and harvested until OD600 ~ 1. K*. pneumoniae 260* strain was then collected by centrifugation, washed once, and resuspended in PBS.

For expression of recombinant proteins (11), *Escherichia coli* BL21 strain (DE) was grown in Luria–Bertani broth containing 40 μg/ml kanamycin up to OD600 ~ 0.5. One liter of bacterial culture was induced with 0.5–1 mM isopropyl-β-D1-thiogalactopyranoside (IPTG) and grown for 4 h at 37°C.

### Cell Shaving of *K. pneumoniae* and Liquid Chromatography–Mass Spectrometry

**Figure 1:**
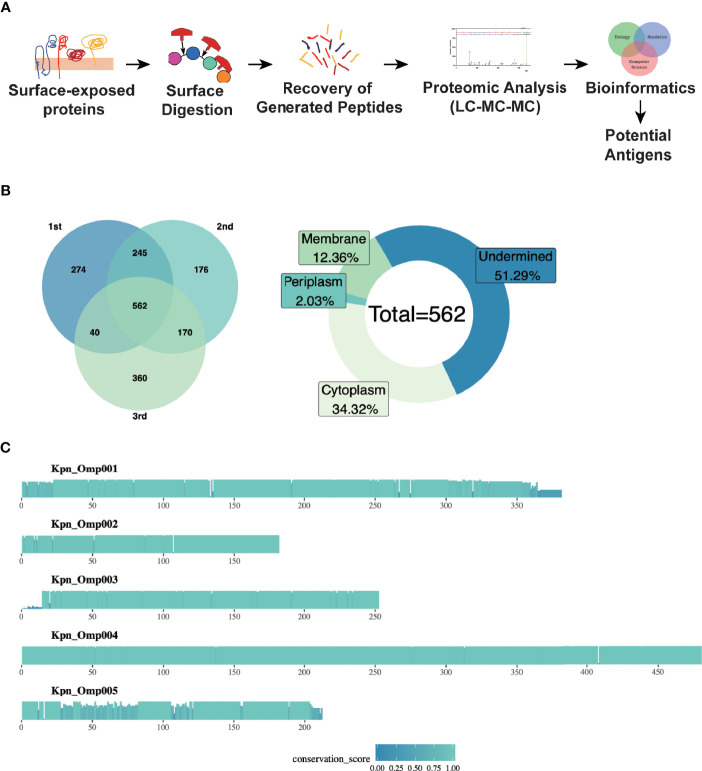
Vaccine antigen gene analysis. **(A)** The workflow for selecting potential antigens combining proteomics with bioinformatics analysis. After “shaving” directly digested the surface protein, process the enzyme digested samples for LC-MS/MS to determine the cut-down proteins. Sequence conservation analysis predicted potential antigens. **(B)** Sequence conservation analysis. Comparing the genomic information of 389 *Klebsiella pneumoniae* strains obtained a conserved core gene database, which contains 562 proteins. The database was then analyzed from the structure information. The predicted surface proteins or secreted proteins were aligned with mass spectral data to exclude non-specific mass spectral data, resulting in the most potential antigens. **(C)** Conservative analysis of KOMPs. The amino acid sequence of these KOMPs (Kpn_Omp001, Kpn_Omp002, Kpn_Omp003, Kpn_Omp004, and Kpn_Omp005) identity ranges from 96% to 100%.

The workflow is shown in **Figure 1A**. Bacterial cells were harvested by centrifugation at 3,500 × *g* for 10 min, at 4°C, and washed three times with PBS, pH 7.4. Cell pellets were resuspended in PBS containing 30% sucrose (pH 7.4) as trypsin incubation buffer. Tryptic digestions were carried out with porcine sequencing grade modified trypsin (Promega) for different times at 37°C. Samples of the bacterial suspension were taken pre- and post-protease digestion to determine colony-forming units (CFUs). Bacterial cells were removed by centrifugation (3500 × *g*, 10 min, 4°C). The supernatants (the “surfome” containing the peptides) were filtered through 0.22-μm-pore-size filters to remove any remaining bacterial cells. “Surfome” was digested with 2 μg of trypsin overnight at 37°C with agitation. Then, the samples were desalted prior to MS and eventually eluted in 70% ACN and 0.1% formic acid. Afterwards, the sample was then placed in SpeedVac to allow the solvent to evaporate completely, and the resulting dry sample was stored at −20°C for use. The solvent was completely vaporized by SPEEDVAC, and the resulting dry sample was stored at −20°C for mass spectrometric (MS) anylsis. After mass spectrometry (MS) conducted by MS machine (Orbitrap Fusion™ Tribrid™ Mass Spectrometer, thermo), the database of the *K. pneumoniae* strains from UniProt was used. The identification of the MS results was performed with Thermo Proteome Discoverer (version 1.4.1.14).

### Vaccine Antigen Gene Analysis

Complete genomic sequences of 389 K*. pneumoniae* strains were downloaded from GenBank (ftp://ftp.ncbi.nlm.nih.gov/genomes/, accessed: 11/18/2019). The potential KOMP sequences were then aligned to genomic sequences with the help of blast from BLAST (12); all 389 genomes obtained at least one protein sequences with at least 60% identity and 70% coverage. Multiple sequence alignment (MSA) was executed by MUSCLE with default parameter using the sequences obtained above (13). The Valdar scoring method was applied for the calculation of residues conservation score as it incorporates sequence redundancy and alignment gaps; calculation was performed on Jalview Version 2 using online method AAcon (Amino Acid Conservation) (14, 15).

### Cloning, Expression, and Purification of Antigens

DNA sequences of KOMPs were amplified by PCR using genomic DNA of *K. pneumoniae* 260 strain as template. The PCR products were then cloned into pET28a vector. Next, the recombinant plasmids were transformed into *E. coli* BL21 strain (DE). A single transformed BL21 clone was subjected to induction with IPTG followed by testing for protein expression by SDS-PAGE analysis. Bacteria were harvested by centrifugation and resuspend in binding buffer (Tris–HCl, pH 8.0, 20 mM imidazole) followed by sonication. After centrifugation at 15,000 × *g* for 30 min at 4°C, the soluble cell extracts were collected and filtered using a 0.22-μm membrane. The samples were then loaded on a nickel-activated chelating Sepharose column (GE Healthcare). After washing, the bound proteins were eluted with elution buffer (Tris–HCl, pH 8.0, 250 mM imidazole). The expression of purified proteins was identified using SDS-PAGE analysis with anti-histidine antibodies. The proteins were subject to endotoxin removal afterwards (Pierce).

### Active Immunization

Five KOMPs formulated with aluminum hydroxide gel adjuvant (Alum, 1 mg/ml) was injected subcutaneously into BALB/c mice (10 per group), respectively. The mice were subjected to booster vaccinations every 2 weeks twice. Alum adjuvant with 25 μg of each purified protein was used to immunize animals. Mice of mock group received an equal amount of Alum adjuvant in PBS. The antibody titers to each antigen in serum samples collected from animals were documented by enzyme-linked immunosorbent assay (ELISA) after each booster vaccination as described elsewhere (16).

### Murine Bacteremia Model

Active (10 days after second booster vaccination) immunized BALB/C mice were challenged with a lethal dose of 1 × 10^9^ CFU *K. pneumoniae* 260 by tail vein intravenous injection. The total volume of the injection was 100 μl. The wellbeing of infected mice was monitored daily for 14 days.

### Murine Pneumonia Model

*K. pneumoniae* 260 was used in the pneumonia model. After anesthesia with ketamine (100 mg/kg) and xylazine (5 mg/kg), BALB/c mice were challenged intranasally with 1 × 10^9^ CFU of *K. pneumoniae* 260. The total volume of the inoculation was 50 μl. The wellbeing of infected mice was monitored daily for 144 h.

### Bacterial Load Assay

Seven days after the third booster vaccination, animals were challenged with a sub-lethal dose, 5 × 10^8^ CFU, of *K. pneumoniae* 260. The lungs, kidneys, and spleens were collected 4 days after the sub-lethal challenge and homogenized. CFUs were enumerated following serial diluting and plating on BHI agar.

### ELISPOT Assay

Mice were sacrificed 7 days after the second booster vaccination. After euthanasia, spleens were collected and single suspensions of splenocytes were obtained (11). Interferon gamma (IFN-γ)-, interleukin 4 (IL-4)-, and interleukin 17A (IL-17A)-producing splenocytes from vaccinated or control mice were analyzed using a cytokine-specific enzyme-linked Immunospot assay (ELISPOT, R&D Systems, United States) as described by the manufacturer. Briefly, splenocytes isolated from immunized mice were plated at a concentration of 1 × 10^5^ cells/well and induced with each antigen alone (0.2 μg/well) or combined (0.1 μg of each antigen/well) in triplicate and incubated for 20 h at 37°C. Ionomycin (1 μg/ml, Sigma, USA) and phorbol myristate acetate (PMA, 50 ng/ml, Sigma) were used as positive controls. Splenocytes from unstimulated, immunized mice and RPMI 1640-treated splenocytes were used as negative controls. After the cells were decanted, biotinylated primary monoclonal antibodies were added to each well and the plates were incubated for 1 h at 37°C. The plates were incubated with streptavidin–HRP conjugate for 1 h at 37°C and subjected to color development with TMB solution. Finally, the spots were enumerated using an Immunospot analyzer.

### Opsonophagocytosis Killing Assay

The opsonophagocytosis killing assay (OPKA) was performed as previously described (17). Human promyelocytic leukemia cells HL-60 were differentiated into phagocytes using 0.8% DMF. *K. pneumoniae* 260 grown overnight were washed once in PBS and resuspended in HBSS buffer (Ca^2+^/Mg^2+^). The bacteria were incubated with heat-activated mouse antiserum against different KOMPs at 4°C for 20 min. Differentiated HL-60 cells were distributed at 3.7 × 10^6^/well with MOI ~ 50:1 in the presence of 10% (v/v) rabbit complement. Following incubation at 37°C for 1 h with agitation at 600 rpm, samples were plated on BHI agar plates for CFU enumeration.

### Animal Ethics

BALB/c mice were supplied by the Laboratory Animal Unit (LAU) of the University of Hong Kong. All mouse experiments were approved by the Committee on the Use of Live Animal in Teaching and Research of the University of Hong Kong (CULATR 4493-17).

### Statistical Analysis

At least two independent experiments, run under the same conditions, were performed for all studies. All data were analyzed in GraphPad prism 8.0 software (GraphPad Software Inc., CA, USA). For the blood infection and pneumonia models, statistical significance was assessed with the log-rank (Mantel-Cox) analysis. The Student’s paired *t*-test was used to analyze the statistical significance of OPKA experiments and bacterial load measurements.

## Results

### Identification and Expression of *K. pneumoniae* Outer Membrane Proteins

Both the “shaving” experiments and the MS experiments were repeated three times independently. After comparing the data from all three experiments, only peptides that were detected in all three experiments were accepted. A total of 562 protein candidates were obtained. Among the 562 proteins, there were 12.36% of membrane proteins, 2.03% of periplasm proteins, 34.32% of cytoplasm proteins, and 51.29% of unknown protein (**Figure 1B**). After analyzing 389 K*. pneumoniae* genome sequences available in NCBI databases, we obtained five proteins that are highly conserved in all strains selected. The amino acid sequence identity ranges from 96% to 100% (**Figures 1B, C**); we name them Kpn_Omp001 (GenBank: QDX70306.1), Kpn_Omp002 (GenBank: QEX42815.1), Kpn_Omp003 (GenBank: QER79328.1), Kpn_Omp004 (GenBank: QEX41443.1), and Kpn_Omp005 (GenBank: QDX64613.1). Recombinant KOMPs were expressed in *E. coli* BL21 and purified using a three-step chromatography strategy. Results indicated that the majority were KOMPs expressed in the soluble form in a high yield (>90%).

### Generation of Antibody-Mediated Immune Responses

**Figure 2:**
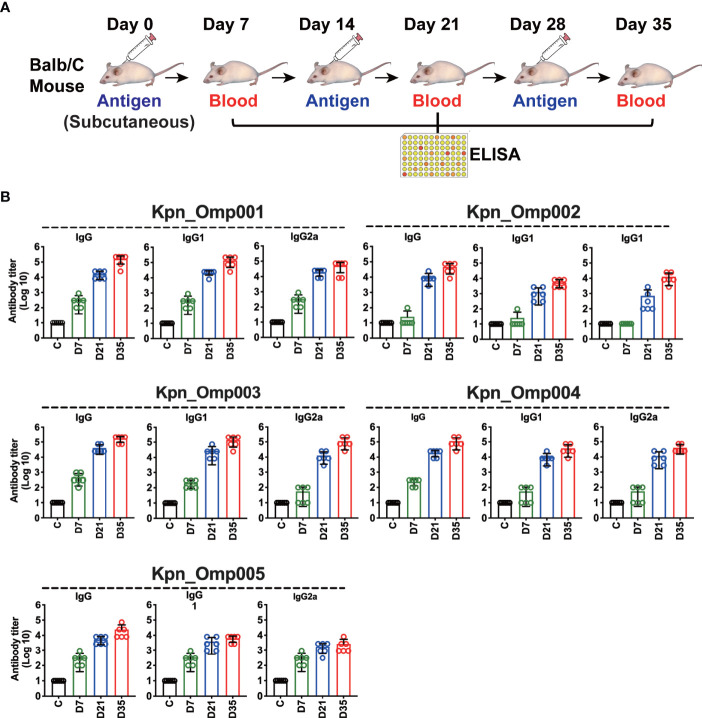
Specific antibody responses in immunized mice. **(A)** Balb/c mice were immunized with the five KOMPs individually or PBS, and antibody titers were determined by enzyme-linked immunosorbent assay (ELISA). **(B)** Specific IgG/IgG1/IgG2a antibody responses in mouse sera were collected at 7 days after the third vaccination of the five KOMPs. The experiment was repeated at least twice.

To measure the antibody-mediated responses of KOMPs (Kpn_Omp001, Kpn_Omp002, Kpn_Omp003, Kpn_Omp004, and Kpn_Omp005), Balb/c mice were immunized with KOMPs individually or PBS according to **Figure 2A**, and KOMP-specific antibody titers were determined by ELISA. High levels of IgG, IgG1, and IgG2a were observed against target antigens in all immunized mice at days 7, 21, and 35. Antibody production after two boost immunizations was considerably higher than that with one immunization. Analysis of the IgG isotype revealed that vaccination with any of the five KOMPs induced both Th1- and Th2-associated antigen-specific IgG2a and IgG1 antibody responses (**Figure 2B**). IgG, IgG1, and IgG2a specific for five KOMPs were not detected in the serum of mice mock immunized with PBS plus AHG.

### Protective Immunity Against Acute *K. pneumoniae* Infection

**Figure 3:**
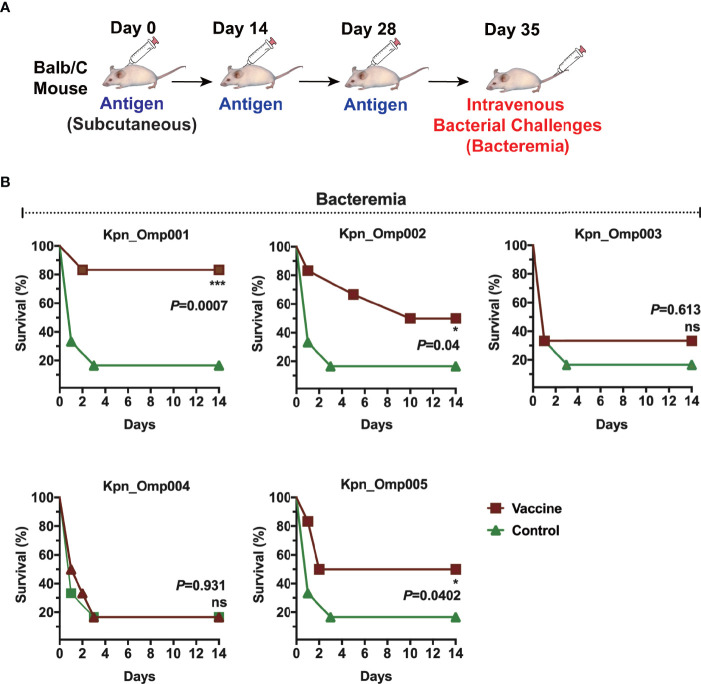
Murine bacteremia model. **(A)** Balb/c mice were immunized with the KOMPs individually or PBS. Seven days after the second booster vaccination, different doses of *K pneumoniae* 260 in 100 μl of PBS were injected intravenously into mice. **(B)** The survival rate of murine bacteremia model was observed every 12 h for 14 days. *p < 0.05, ***p < 0.001. ns, p > 0.05.

One of the criteria for selecting these KOMPs as vaccine candidates was their ability to induce protection in one or more animal models of *K. pneumoniae* infection. Mice were immunized with each KOMP formulated with aluminum hydroxide. After immunization, mice were challenged intravenously (i.v.) with a lethal dose of 1 × 10^9^ CFU *K. pneumoniae* 260 (**Figure 3A**). The survival of mice was recorded in a 14-day period. In the bacteremia model, immunization with Kpn_Omp001, Kpn_Omp002, and Kpn_Omp005 resulted in significantly increased survival rate compared with the control mice immunized with only adjuvant (Alum, mock) (**Figure 3B**). More than 80% (*p* = 0.0007) of Kpn_Omp001, 50% (*p* = 0.04) of Kpn_Omp002, and 50% (*p* = 0.0402) of Kpn_Omp005 immunized mice survived over 14 days after lethal challenge. The survival rate of mice immunized with Kpn_Omp003 and Kpn_Omp004 did not show any protection compared with the control mice (**Figure 3B**).

**Figure 4:**
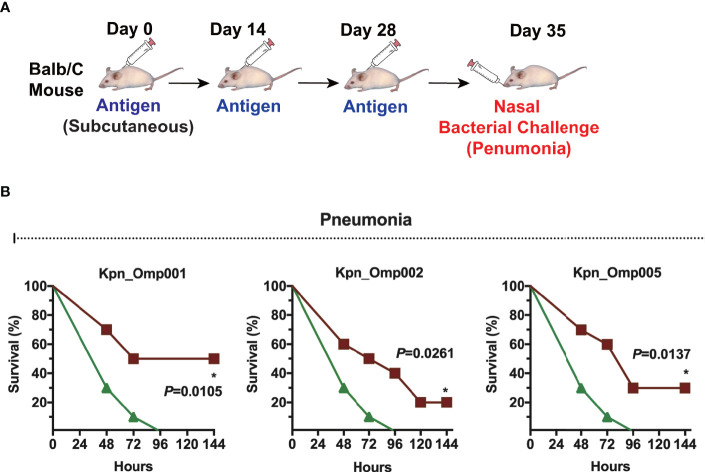
Murine pneumonia model. **(A)** Balb/c mice were immunized with the KOMPs individually or PBS. Seven days after the second booster vaccination, different doses of *K pneumoniae* 260 in 50 μl of PBS were injected intranasally into mice. **(B)** The survival rate of murine pneumonia model was observed for 144 h. *p < 0.05.

Then, we evaluated the efficacy of Kpn_Omp001, Kpn_Omp002, and Kpn_Omp005 in the pneumonia model (**Figure 4A**). The survival of immunized and control mice was evaluated for a 144-h period after nasal challenge of lethal dose of *K. pneumoniae* strain 260. The survival rate of mice immunized with Kpn_Omp001 (50%, *p* = 0.0105), Kpn_Omp002 (20%, *p* = 0.0261), or Kpn_Omp005 (30%, *p* = 0.0137) was always significantly superior to that observed in mock (**Figure 4B**).

**Figure 5:**
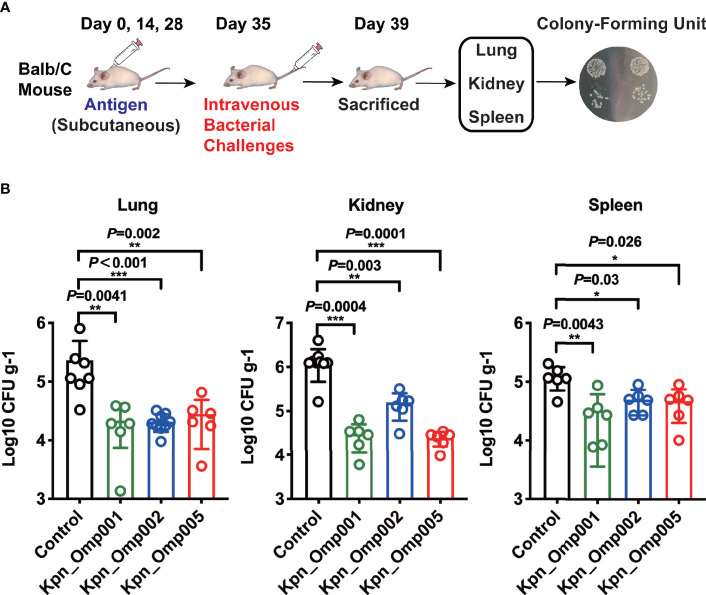
Bacterial load assay. **(A, B)** Balb/c mice were immunized with the Kpn_Omp001, Kpn_Omp002, and Kpn_Omp005 individually or PBS, and they were injected with 5 × 10^8^ CFU of *K pneumoniae* 260 in 7 days after the second booster vaccination. The lungs, kidneys, and spleens were collected 4 days after the sub-lethal challenge and homogenized. CFUs were enumerated following serial diluting and plating on BHI agar. *p < 0.05, **p < 0.01, ***p < 0.001.

In addition, the bacteria load recovered from different organs in mice after sub-lethal dose of intravenous challenge of *K. pneumoniae* strain was counted (**Figure 5A**). As shown in **Figure 5B**, immunization with Kpn_Omp001, Kpn_Omp002, and Kpn_Omp005 resulted in a significant reduction of bacterial load when compared with the control mice immunized with alum alone. The reduction of bacteria load was most significant in kidneys. Reduction of CFUs varied from a minimum of 1 to a maximum of 2 logs.

### Activation of Cell-Mediated Immunity

**Figure 6:**
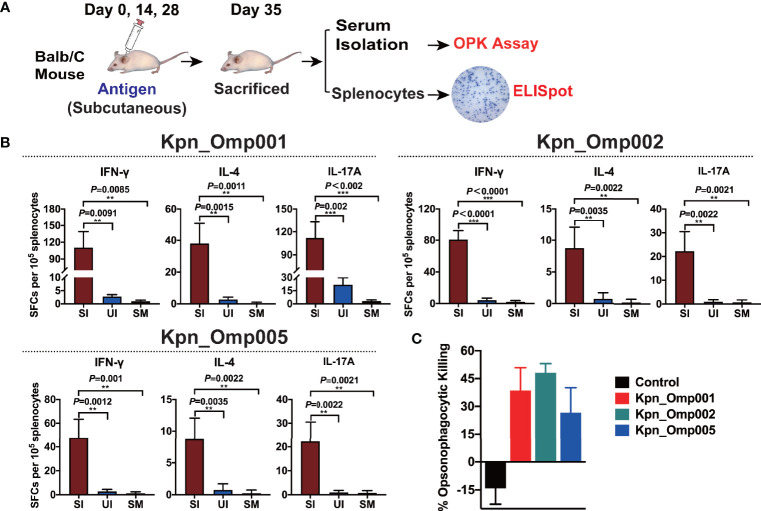
ELISPOT assay and opsonophagocytosis killing assay. **(A)** Balb/c mice were immunized with the Kpn_Omp001, Kpn_Omp002, and Kpn_Omp005 individually or PBS, and mice were sacrificed 7 days after the second booster vaccination. After euthanasia, spleens were collected and a single suspension of splenocytes was obtained for cytokine-specific enzyme-linked Immunospot assay (ELISPOT assay), and the serum was isolated for the opsonophagocytosis killing assay. **(B)** Interferon gamma (IFN-γ), interleukin 4 (IL-4), and interleukin 17A (IL-17A)-producing splenocytes from vaccinated or control mice were analyzed using ELISPOT assay. SI: Immunized mice stimulated with KOMP, UI: Unstimulated immunized mice, SM: Mock mice stimulated with KOMP. **(C)** The opsonophagocytosis killing assay. The bacteria were incubated with heat-activated mouse antiserum against different KOMPs at 4°C for 20 min, differentiated HL-60 cells and rabbit complement co-incubated at 37°C for 1 h with agitation at 600 rpm, and samples were plated on BHI agar plates for CFU enumeration. **p < 0.01, ***p < 0.001.

To evaluate T-cell responses generated by these three potential KOMPs, we measured KOMP-specific IFN-γ-, IL-4-, and IL-17A-secreting splenocytes of Balb/C mice after *ex vivo* re-stimulation, using ELISpot assay (**Figure 6A**). These three potential KOMPs (Kpn_Omp001, Kpn_Omp002, or Kpn_Omp005) elicited a significant increase in the number of IFN-γ-, IL-4-, and IL-17A-secreting splenocytes (**Figure 6B**). These findings suggest that immunization with Kpn_Omp001, Kpn_Omp002, or Kpn_Omp005 induces strong antigen-specific Th1, IFN-γ-producing T-cell response in addition to Th2 and Th17 responses.

### Opsonophagocytosis Assays

Next, we performed if its antiserum could facilitate opsonophagocytosis of *K. pneumoniae*. The antiserum samples were incubated with *K*. *pneumoniae* 260 and differentiated HL60 cells in the presence of rabbit complement. As shown in **Figure 6C**, serum from alum-immunized mouse did not mediate bacterial killing. Likewise, bacterial killing was not facilitated without HL-60 cells or active rabbit complement. On the contrary, HL-60 cells killed over 40%, 50%, or 30% of *K. pneumoniae* with the anti-Kpn_Omp001, anti-Kpn_Omp002, or anti-Kpn_Omp005 serum. These results suggest that humoral immunity may confer the protective effect of *K. pneumoniae* anti-Kpn_Omp001, anti-Kpn_Omp002, or anti-Kpn_Omp005 vaccination.

## Discussion

*K. pneumoniae* is the most common cause of nosocomial respiratory tract and premature intensive care infections and the second most frequent cause of Gram-negative bacteremia (3). However, passive immunization and immunoenhancers were shown to not be practical for the prevention of *K. pneumoniae* infections (18). Vaccination is considered to be the most cost-effective and effective approach to prevent infectious diseases. Among these efforts, capsular polysaccharide was targeted as a vaccine antigen, but the variable capsular polysaccharide serotypes limited their use in vaccine production (19). The strategy of targeting non-capsular protein antigens revealed that outer membrane proteins are protective antigens in the immune response evoked by *K. pneumoniae.* No single conserved antigen has been identified as a candidate to accelerate for vaccine development.

In the present study, we aimed to select and evaluate the potential KOMPs as new vaccine candidates to prevent lethality by *K. pneumoniae* infection. The rapid development of proteomics has provided us with a lot of assistants on research (20). In the antigen screening process, we mainly combined proteomics with bioinformatics analysis methods to find reliable potential antigens. First, we used the most advanced biochemical research method, “shaving”, which directly digested the protein on the surface of live pathogen cells with trypsin, and further processed the enzyme digested samples for LC-MS/MS to determine the cut-down proteins (21). Ideally, we cut off the outer surface of the outer membrane protein of the pathogen, so that the proteins that can be easily cut out also suggest that they are in a more easily exposed position, which is more suitable for antigen-receptor binding (21, 22). However, there are still specific problems by this means alone, for example, intracellular proteins released by cracked bacteria cells can also be cleaved by enzymes. Therefore, our refined approach is to first compare the genomic information of many different strains of the pathogen by bioinformatics and obtain a conserved core gene database, and then predict all the surface protein and secreted protein. The part predicted to be surface proteins and secreted proteins are then aligned with our mass spectral data to exclude non-specific mass spectral data, ultimately resulting in more reliable potential antigens. After analyzing 389 K*. pneumoniae* genome sequences available in NCBI databases, we obtained five KOMPs that are highly conserved in all strains selected. The amino acid sequence identity ranges from 96% to 100%, and we name them Kpn_Omp001, Kpn_Omp002, Kpn_Omp003, Kpn_Omp004, and Kpn_Omp005.

We then thoroughly evaluated the humoral and cellular immunity they induced in BALB/c mice as well as their protection against *K. pneumoniae* infection. These five KOMPs induce high antigen-specific IgG, IgG1, and IgG2a titer. However, just three KOMPs (Kpn_Omp001, Kpn_Omp002, and Kpn_Omp005) were able to induce a protective immune response against two *K. pneumoniae* infection models. Low bacterial load of *K. pneumoniae* was recovered from the different organs such as lungs, kidney, and spleen of vaccinated mice compared with the control group, indicating the protective efficacy of the KOMPs vaccine. These protective effects were accompanied by the involvement of different immune responses induced by KOMPs, which included KOMP-specific IgG, IgG1, and IgG2a, and IFN-γ-, IL4-, and IL17A-mediated immune responses.

Given the antigenic diversity between *K. pneumoniae* strains (7), finding conserved antigens will maximize the chances of success in vaccine development or in development of alternative prevention strategies such as monoclonal antibodies. The study findings suggest that Kpn_Omp001, Kpn_Omp002, and Kpn_Omp005 are novel vaccine candidates for the prevention of *K. pneumoniae* infections.

## Data Availability Statement

The original contributions presented in the study are included in the article/supplementary material. Further inquiries can be directed to the corresponding authors.

## Ethics Statement

All mouse experiments were approved by the Committee on the Use of Live Animal in Teaching and Research of the University of Hong Kong (CULATR 4493-17).

## Author Contributions

B-ZZ and J-DH designed the experiment. B-ZZ, DH, and YD performed the experiments. LX, XW, JH, S-ZX, WL, J-PC, MJ, MZ, and QL participated in the study. B-ZZ and J-DH wrote the manuscript. B-ZZ and J-DH analyzed the data. K-YY and ML provided essential reagents and critical comments. All authors contributed to the article and approved the submitted version.

## Funding

This work was supported by the National Key Research and Development Program (2018YFA0903000), the Shenzhen Peacock project (KQTD2015033117210153), and Health and Medical Research Fund (HMRF, HKM-15-M09).

## Conflict of Interest

The authors declare that the research was conducted in the absence of any commercial or financial relationships that could be construed as a potential conflict of interest.

## Publisher’s Note

All claims expressed in this article are solely those of the authors and do not necessarily represent those of their affiliated organizations, or those of the publisher, the editors and the reviewers. Any product that may be evaluated in this article, or claim that may be made by its manufacturer, is not guaranteed or endorsed by the publisher.

## References

[1] Martin RM Bachman MA. Colonization, Infection, and the Accessory Genome of Klebsiella Pneumoniae. Front Cell Infect Microbiol (2018) 8:4. doi: 10.3389/fcimb.2018.00004 29404282 PMC5786545

[2] Jun JB. Klebsiella Pneumoniae Liver Abscess. Infect Chemother (2018) 50:210–8. doi: 10.3947/ic.2018.50.3.210 PMC6167513 30270580

[3] Bengoechea JA Sa Pessoa J. Klebsiella Pneumoniae Infection Biology: Living to Counteract Host Defences. FEMS Microbiol Rev (2019) 43:123–44. doi: 10.1093/femsre/fuy043 PMC6435446 30452654

[4] Seifert H Blondeau J Dowzicky MJ. In Vitro Activity of Tigecycline and Comparators (2014-2016) Among Key WHO ’Priority Pathogens’ and Longitudinal Assessment (2004-2016) of Antimicrobial Resistance: A Report From the T.E.S.T. Study. Int J Antimicrob Agents (2018) 52:474–84. doi: 10.1016/j.ijantimicag.2018.07.003 30012439

[5] Govindaraj Vaithinathan A Vanitha A. WHO Global Priority Pathogens List on Antibiotic Resistance: An Urgent Need for Action to Integrate One Health Data. Perspect Public Health (2018) 138:87–8. doi: 10.1177/1757913917743881 29465015

[6] Lundberg U Senn BM Schuler W Meinke A Hanner M. Identification and Characterization of Antigens as Vaccine Candidates Against Klebsiella Pneumoniae. Hum Vaccin Immunother (2013) 9:497–505. doi: 10.4161/hv.23225 23250007 PMC3891705

[7] Follador R Heinz E Wyres KL Ellington MJ Kowarik M Holt KE. The Diversity of Klebsiella Pneumoniae Surface Polysaccharides. Microb Genom (2016) 2:e000073. doi: 10.1099/mgen.0.000073 28348868 PMC5320592

[8] Jahns AC Rehm BH. Relevant Uses of Surface Proteins–Display on Self-Organized Biological Structures. Microb Biotechnol (2012) 5:188–202. doi: 10.1111/j.1751-7915.2011.00293.x 21906264 PMC3815779

[9] Stuber JC Kast F Pluckthun A. High-Throughput Quantification of Surface Protein Internalization and Degradation. ACS Chem Biol (2019) 14:1154–63. doi: 10.1021/acschembio.9b00016 31050891

[10] Asif AR Oellerich M Amstrong VW Riemenschneider B Monod M Reichard U. Proteome of Conidial Surface Associated Proteins of Aspergillus Fumigatus Reflecting Potential Vaccine Candidates and Allergens. J Proteome Res (2006) 5:954–62. doi: 10.1021/pr0504586 16602703

[11] Zhang BZ Hua YH Yu B Lau CC Cai JP Zheng SY. Recombinant ESAT-6-Like Proteins Provoke Protective Immune Responses Against Invasive Staphylococcus Aureus Disease in a Murine Model. Infect Immun (2015) 83:339–45. doi: 10.1128/IAI.02498-14 PMC4288882 25368117

[12] Camacho C Coulouris G Avagyan V Ma N Papadopoulos J Bealer K. BLAST+: Architecture and Applications. BMC Bioinf (2009) 10:421. doi: 10.1186/1471-2105-10-421 PMC2803857 20003500

[13] Madeira F Park YM Lee J Buso N Gur T Madhusoodanan N. The EMBL-EBI Search and Sequence Analysis Tools APIs in 2019. Nucleic Acids Res (2019) 47:W636–41. doi: 10.1093/nar/gkz268 PMC6602479 30976793

[14] Valdar WS. Scoring Residue Conservation. Proteins (2002) 48:227–41. doi: 10.1002/prot.10146 12112692

[15] Waterhouse AM Procter JB Martin DM Clamp M Barton GJ. Jalview Version 2–a Multiple Sequence Alignment Editor and Analysis Workbench. Bioinformatics (2009) 25:1189–91. doi: 10.1093/bioinformatics/btp033 PMC2672624 19151095

[16] Zhang BZ Cai J Yu B Xiong L Lin Q Yang XY. Immunotherapy Targeting Adenosine Synthase A Decreases Severity of Staphylococcus Aureus Infection in Mouse Model. J Infect Dis (2017) 216:245–53. doi: 10.1093/infdis/jix290 PMC5853884 28633319

[17] Deng J Wang X Zhang BZ Gao P Lin Q Kao RYT. Broad and Effective Protection Against Staphylococcus Aureus Is Elicited by a Multivalent Vaccine Formulated With Novel Antigens. mSphere (2019) 4(5):e00362–19. doi: 10.1128/mSphere.00362-19 PMC6731528 31484738

[18] Sun WS Syu WJ Ho WL Lin CN Tsai SF Wang SH. SitA Contributes to the Virulence of Klebsiella Pneumoniae in a Mouse Infection Model. Microbes Infect (2014) 16:161–70. doi: 10.1016/j.micinf.2013.10.019 24211873

[19] Ahmad TA El-Sayed LH Haroun M Hussein AA El Ashry el SH. Development of Immunization Trials Against Klebsiella Pneumoniae. Vaccine (2012) 30:2411–20. doi: 10.1016/j.vaccine.2011.11.027 22100884

[20] Sun L Rasmussen PK Bai Y Chen X Cai T Wang J. Proteomic Changes of Klebsiella Pneumoniae in Response to Colistin Treatment and crrB Mutation-Mediated Colistin Resistance. Antimicrob Agents Chemother (2020) 64. doi: 10.1128/AAC.02200-19 PMC7269499 32229491

[21] Dreisbach A Wang M van der Kooi-Pol MM Reilman E Koedijk DGAM Mars RAT. Tryptic Shaving of Staphylococcus Aureus Unveils Immunodominant Epitopes on the Bacterial Cell Surface. J Proteome Res (2020) 19:2997–3010. doi: 10.1021/acs.jproteome.0c00043 32529827

[22] Solis N Larsen MR Cordwell SJ. Improved Accuracy of Cell Surface Shaving Proteomics in Staphylococcus Aureus Using a False-Positive Control. Proteomics (2010) 10:2037–49. doi: 10.1002/pmic.200900564 20217865

